# Phosphoinositides Signaling and Epithelial-to-Mesenchymal Transition: Putative Topic for Basic Toxicological Research

**DOI:** 10.5487/TR.2008.24.1.001

**Published:** 2008-03-01

**Authors:** Chang Ho Lee

**Affiliations:** Department of Pharmacology and Biomedical Science, College of Medicine, Hanyang University, Sungdong-gu, Seoul, 133-791 Korea

**Keywords:** PIP kinases, PIPKIγ661, E-cadherin, EMT, Adherence Junction

## Abstract

Ptdlns(4,5)P_2_ is a key cellular phosphoinositide that localizes in separate and distinctive pools in subcellular membrane and vesicular compartments. In membranes, Ptdlns(4,5)P_2_ acts as a precursor to second messengers and is itself a main signaling and targeting molecule. Specific subcellular localization of type I PIP kinases directed by interacting with specific targeting module differentiates Ptdlns(4,5)P_2_ production in a spatial and temporal manner. Several lines of evidences support the idea that Ptdlns(4,5)P_2_ is generated in very specific pools in a spatial and temporal manner or by feeding Ptdlns(4,5)P_2_ directly to effectors. In this concept, the interaction of PIPKI isoforms with a specific targeting module to allow precise subcellular targeting modulates highly specific Ptdlns(4,5)P_2_ synthesis and channeling overall effectors. For instance, localization of PIPKIγ661 to focal adhesions by an interaction with talin results in spatial and temporal production of Ptdlns(4,5)P_2_, which regulates EGF-stimulated directional cell migration. In addition, Type lγ PIPK is targeted to E-cadherin in cell adherence junction and plays a role in controlling dynamics of cell adherence junction and endocytosis of E-cadherin. Characterizing how PIP kinase isoforms are regulated by interactions with their targeting modules, as well as the mechanisms by which their product, Ptdlns(4,5)P_2_, exerts its effects on cellular signaling processes, is crucial to understand the harmonized control of numerous cellular signaling pathways. Thus, in this review the roles of the Ptdlns(4)P(5) kinases and Ptdlns(4,5)P_2_ were described and critically reviewed in terms of regulation of the E-cadherin trafficking, cell migration, and formation of cell adherence junction which is indispensable and is tightly controlled in epithelial-to-mesenchymal transition process.

***Phosphoinositide messengers and PIP kinases.*** It is well known that PtdIns(4,5)$P_2$ is a major cellular phosphoinositide that exists in discrete and compartmentalized pools in subcellular membrane and vesicular structures. In membranes, PtdIns(4,5)$P_2$ acts as a precursor to second messengers and is itself a key signaling and targeting molecule (Niggli, 2005). Distinct PtdIns(4,5)$P_2$ pools are involved in modulating a number of cellular activities such as cell motility (Golub and Caroni, 2005; Insall and Weiner, 2001; Kisseleva *et al.*, 2005), gating of ion channels (Hilgemann and Ball, 1996; Suh and Hille, 2005), exocytosis (Aikawa and Martin, 2003; Aoyagi *et al.*, 2005; Gong *et al.*, 2005) and vesicular trafficking (Downes *et al.*, 2005; Martin, 2001).

PIP kinase family are classified into two subtypes (type I and II), which act to produce PtdIns(4,5)$P_2$ from distinctive substrate pools. Type I PIP kinases (PIPKI) utilize PtdIns(4)P as a substrate (Rameh *et al.*, 1997), while Type II PIP kinases (PIPKII) utilize PtdIns(5)P (Rameh *et al.*, 1997; Zhang *et al.*, 1997). PIPKs also use PtdIns3P as a substrate, with lower *in vitro* efficacy, and, interestingly, produce PtdIns(3,4,5)$P_3$ by a concerted double phosphorylation mechanism (Zhang *et al.*, 1997). The role of PtdIns3P as substrate for production of PtdIns(3,4,5)$P_3$ by PIPK *in vivo* has not yet been characterized. The type I and II PIP kinase subfamilies consist of three major isoforms, α, β, and γ, and these isoforms themselves have a number of splice variants (Boronenkov *et al.*, 1998; Ishihara *et al.*, 1996, 1998; Itoh *et al.*, 1998; Loijens and Anderson, 1996). The nomenclature for PIPKIα and PIPKIβ are switched between humans and mice (Ishihara *et al*., 1998). These PIPK isotypes synthesize PtdIns(4,5)$P_2$ in diverse subcellular locations and are key regulators of PtdIns(4,5)$P_2$-mediated cellular signaling events. Distinct substrate specificity of PIPK isotypes are determined by the activation loop. The activation loops of types I and II PIP kinases differ critically in only one amino acid residue, a glutamate (type I's) or an alanine (type II's), indicating that the substrate binding pocket has a high degree of specificity (Kunz *et al*., 2000). Substrate specificity given by the distinctive activation loop of PIP kinase subfamilies allocate for non-redundant physiological pathways to regulate subcellular PtdIns(4,5)$P_2$ levels.

Specific localization of type I PIP kinases by interacting with unique targeting module defines a spatial and temporal production of PtdIns(4,5)$P_2$ in distinct subcellular compartments (Kunz *et al*., 2000). Several lines of evidences suggest that spatial and temporal production of PtdIns(4,5)$P_2$ is a key process for regulating a number of distinct subcellular events. Each members of the PIP kinases subfamilies have been shown to be downstream effectors in small G-protein signaling cascades and in many cases produce cellular PtdIns(4,5)$P_2$ under control of specific small G-protein signaling units (Doughman *et al*., 2003; Oude Weernink *et al*., 2004). Functional interaction of small G-proteins and PIP kinases makes it possible to utilize subcellular phosphoinositide turnover for coordinated organization of the cellular signaling machinery. For instance, the Rho and Arf families of monomeric G-proteins translocate and regulate activities of each members of PIP kinase subfamilies, thereby coordinating production and availability of PtdIns(4,5)$P_2$ at specific subcellular compartments in the cell. These activities are critical for appropriate regulation of actin dynamics and cytoskeletal-dependent functions of the cell (Honda *et al*., 1999; Yang *et al*., 2004). Additionally, the spatial and temporal synthesis of PtdIns(4,5)$P_2$ by PIP kinases is also regulated by a complex regulatory feedback loop involving phospholipase D and its reaction product, phosphatidic acid (Divecha *et al*., 2000; Jenkins *et al*., 1994; Jones *et al*., 2000; Powner *et al*., 2005; Powner and Wakelam, 2002; Yang *et al*., 2004).

Several lines of evidences support the idea that PtdIns(4,5)$P_2$ is generated in highly specific pools in a spatial and temporal manner or by feeding PtdIns(4,5)$P_2$ directly to effectors (Ling *et al*., 2002, 2006, 2007). In this concept, the interaction of PIPKI isoforms with a specific targeting module to allow precise subcellular targeting modulates highly specific PtdIns(4,5)$P_2$ synthesis and channeling overall effectors. For instance, localization of PIPKIγ661 to focal adhesions by an interaction with talin results in spatial and temporal production of PtdIns(4,5)$P_2$, which regulates EGF-stimulated directional cell migration (Sun *et al*., 2007), as well as strengthens the binding of talin to β-integrin (Ling *et al*., 2002). In addition, Type Iγ PIPK is targeted to E-cadherin in cell adherence junction and plays a role in controlling dynamics of cell adherence junction and endocytosis of E-cadherin (Ling *et al*., 2007).

Characterizing how PIP Kinases are regulated by interactions with their targeting modules, as well as the mechanisms by which their product, PtdIns(4,5)$P_2$, exerts its effects on cellular signaling processes, is critical to understand the harmonized control of numerous cellular signaling pathways. Thus, in this review the roles of the PtdIns(4)P(5) kinases and PtdIns(4,5)$P_2$ were described and critically reviewed in terms of regulation of the E-cadherin trafficking, cell migration, and formation of cell adherence junction which is indispensable and is tightly controlled in EMT process.

***Definition of EMT.*** The epithelial-to-mesenchymal transition (EMT) is a cellular process characterized by a loss of the polarized epithelial phenotype with a transition to a mesenchymal or more migratory phenotype. EMT is a normal biological process essential for cell movement and generation of new tissue types during embryonic development. It is a reversible process that occurs when cells lose a polarity and the cell-cell contacts which are formed via adherence junctions, migrate to different subcellular locations, and then revert to their original phenotype via mesenchymal-to-epithelial transition (MET). EMT, if it is deregulated, also plays a role in the pathogenic process such as cancer metastasis and tissue fibrosis (Arias, 2001; Hugo *et al*., 2007; Moustakas and Heldin, 2007; Thiery, 2002).

***Cadherins, adherence junction, and cell polarity.*** Adherence junction, tight junction, and desmosomes support adhesion between neighboring epithelial cells, initiate the assembly of the mechanical cytoskeletal linkage, and facilitate the formation of a polarized epithelial monolayers (Gumbiner, 1996). These junctions allow the separation of apical and basolateral membrane domains that vary in protein and lipid content, and the resultant polarity will be crucial to normal cell function. (Wodarz and Nathke, 2007). In epithelia, adherence junction formation is mediated by homophilic binding of E-cadherin molecules on neighboring cells, which is a calcium-dependent process (Gumbiner, 1996). These interactions connect adjacent cells and promote the assembly of cadherin complex consisting of p120-, β-, and α-catenins, which links E-cadherin complex to the actin cytoskeleton. The E-cadherin-mediated cell adherence junction is thought to serve as an initial cue for the development of apical-basolateral cell polarity (Theard *et al*., 2007; Vega-Salas *et al*., 1987).

There exists a diverse family of cadherin proteins. The expression of different cadherin isoforms during various stages of tissue development promotes proper cell proliferation and differentiation and provides cues for the maintenance of tissue and organ integrity (Carvell *et al*., 2007; Syed *et al*., 2007; Vestweber, 2007). Cadherins maintain adhesive junctions between epithelial, vascular endothelial, and cardiac myocytes (Halbleib and Nelson, 2006; Vestweber, 2007; Zuppinger *et al*., 2000). One specific cadherin isoform, E-cadherin, plays a key role in cellular organization during embryogenesis and organogenesis as well as in morphogenesis and wound healing in mature organ (Halbleib and Nelson, 2006). N-cadherin and VE-cadherin modulate cardiac morphology and vascular genesis, respectively (Vestweber, 2007; Zuppinger *et al*., 2000).

***EMT signals.*** Several soluble factors, such as epidermal growth factor (EGF), hepatocyte growth factor (HGF), fibroblast growth factor (FGF), and transforming growth factor (TGF$\beta$) induce EMT *in vivo* (Radisky, 2005; Thiery, 2002; Willis and Borok, 2007; Zavadil and Bottinger, 2005). *In vitro*, however, these signals are not function-specific and can trigger both EMT and proliferation. EMT is a characteristic feature of cells undergoing proliferation (Arias, 2001; Birchmeier and Birchmeier, 1995; Thiery, 2002; Vernon and LaBonne, 2004). While several signal transduction pathways have been implicated in the regulation of EMT, it is not fully clear as to how this process is initiated and perpetuated.

Diverse cellular signaling events mediated by, in particular, the PI3-kinase, monomeric G-protein Rac and Ras, and protein tyrosine kinases, Fak and Src pathways, can also initiate and modulate EMT (Frame *et al*., 2002; Savagner, 2001; Van Aelst and Symons, 2002). However, these pathways must first disrupt direct cadherin-cadherin cell contacts in adherence junctions. These junctions assemble in sequential steps initiated by a lateral dimerization and homophilic binding of E-cadherin between cells, resulting in weak cell-cell adhesion (Anastasiadis and Reynolds, 2000; Conacci-Sorrell *et al*., 2002; Jamora and Fuchs, 2002; Knust, 2000). In addition, formation of a complex of cytosolic proteins, including catenins and actin binding proteins such as $\alpha$-actinin is required to mediate a linkage to the actin cytoskeleton, which strengthens cell-cell contacts. In epithelial cells, the configuration of strong cell adhesion complexes is controlled by various signaling pathways involving not only those mentioned above, but also the mitogen activated protein kinase (MAP kinase) signaling pathway and transcriptional regulation mediated by Snail and Slug transcription factor (Arias, 2001; Birchmeier and Birchmeier, 1995; Cano *et al*., 2000; Frame *et al*., 2002; Frisch, 1997; Savagner, 2001; Thiery, 2002; Van Aelst and Symons, 2002). Ras-MAP kinase signaling axis can activate two related transcription factor Snail and Slug which are transcriptional repressors of E-cadherin and consequently, expression of Snail and Slug induce a loss of cadherin-mediated intercellular adhesion that is a prerequisite for EMT (Becker *et al*., 2007; Hajra *et al*., 2002).

A characteristic feature of the EMT can be observed in epithelial cell-derived cancers, such as ovarian, breast, colon, and prostate cancers. More than sixty percent of all cancers are derived from epithelial cells (Hanahan and Weinberg, 2000). The loss of E-cadherin-mediated cell-cell contacts in epithelial cells leads to a loss of polarization and is a key indicator of transition from the epithelial to the mesenchymal phenotype (Arias, 2001; Birchmeier and Birchmeier, 1995; Frame *et al*., 2002; Frisch, 1997; Hanahan and Weinberg, 2000; Savagner, 2001; Thiery, 2002; Van Aelst and Symons, 2002). Clinically, the loss of cell surface E-cadherin and the gain of N-cadherin is a convincing marker of tumor metastasis as well as poor patient prognosis. During tumor progression, the expression of E-cadherin is suppressed or inactivated by Snail or Slug-dependent transcriptional mechanisms (Nelson and Nusse, 2004). In addition, control of E-cadherin turnover can modulate its role in adherence junction. For instance, the loss of E-cadherin from cell adherence junctions is initiated by tumor promoting EMT signaling pathways involving Src, Ras, and growth factors (Birchmeier and Birchmeier, 1995; Frame *et al*., 2002; Frisch, 1997; Hanahan and Weinberg, 2000; Savagner, 2001; Thiery, 2002; Van Aelst and Symons, 2002).

Cellular interaction with extracellular matrix components also influences on EMT processes. In particular, there are roles for integrin signaling in EMT, specifically involving $\beta$1 and $\beta$3 integrins. Integrins are heterodimeric extracellular matrix (ECM) receptors. These receptors consist of one $\alpha$- and one $\beta$-integrin subunit and mediate epithelial cell binding to the ECM through focal adhesion, leading to diverse cell signaling cascades that regulate cell migration, proliferation, cell survival and cellular phenotype (Hynes, 2002). Integrin-linked kinase (ILK) mediates integrin signaling triggered by extracellular matrix molecules and other growth factors by interacting with the cytoplasmic domain of $\beta$1 and $\beta$3

integrin subunits (Oloumi *et al*., 2004). Activation of ILK in epithelial cells results in disruption of cell-extracellular matrix and intercellular adhesions through down-regulation of E-cadherin expression, and nuclear buildup of β-catenin, suggesting that changing integrin signaling can modulate the strength of intercellular adhesions, and thus controlling EMT process (Oloumi *et al*., 2004).

***PIP kinase regulation of cell migration, E-cadherin trafficking, and EMT.*** Several lines of evidences suggest a role of PIP Kinase in EMT via the spatial and temporal generation of PtdIns(4,5)$P_2$ since cell migration and the modulation of adherence junction and E-cadherin trafficking are modulated by PtdIns(4,5)$P_2$-dependent mechanisms (Ling *et al*., 2002, 2006, 2007; Sun *et al*., 2007). Cell migration requires the highly coordinated processes including focal adhesion formation and turnover as well as dynamic changes in cytoskeletal rearrangements (Horwitz and Parsons, 1999; Webb *et al*., 2002). The sequential phases of cell movement include extension of a protrusion at the leading edge of the cell, formation of stable attachments near the leading edge of the protrusion, and then release of attachment and retraction at the trailing edge of the cells, thus providing driving force for cell movement (Webb *et al*., 2002). Actin polymerization dynamics is a well-established underlining mechanism in the formation of leading edge protrusion (DesMarais *et al*., 2005). Many observations indicate that PtdIns(4,5)$P_2$ is a key signaling molecule in the regulation of the assembly of the cell migratory machinery. PtdIns(4,5)$P_2$ regulates dynamics of the actin rearrangement by interacting with diverse actin binding proteins such as α-actinin, WASP/N-WASP, gelsolin, cofilin, and villin (Fawcett and Pawson, 2000; Lanier and Gertler, 2000; Ling *et al*., 2006; Niggli, 2005). PtdIns(4,5)$P_2$ has also been proposed to regulate the dynamics of focal adhesion assembly by interacting with talin, vinculin, ezrin/radixin/moesin, calpain, and other proteins involved in focal adhesion formation (Ling *et al*., 2006; Niggli, 2005). In addition, microtubules facilitate cell migration by being captured and stabilized at the leading edge (Golub and Caroni, 2005) and by triggering the disassembly of focal adhesions (Tilghman *et al*., 2005). Actin-myosin contractility is involved for retracting rear edge of the cells (Yam *et al*., 2007).

The other critical modulators of actin rearrangement are the monomeric G-proteins Rac, Cdc42, Rho, and Arf (Hall, 1998; Moss and Vaughan, 1998; Nobes and Hall, 1995; Ridley and Hall, 1992; Ridley *et al*., 1992; Symons, 1995, 1996; Van Aelst and D'Souza-Schorey, 1997; Zigmond, 1996). These small G-proteins modulate the actin cytoskeleton in membrane ruffle assembly, the assembly of filopodia, lamellipodia, actin stress fibers, and focal adhesions. Phosphoinositide signaling molecule, such as PtdIns(4,5)$P_2$, regulate the activities of these small G-protins in direct and indirect ways (Brown *et al*., 1998; Donaldson and Jackson, 2000; Kam *et al*., 2000; Parker, 1995). For example, membrane ruffling requires harmonization between PIPKIα and Rac signaling processes. Rac1 has been shown to be associated with PIPKIα, forming a Rac1-PIPKIα signaling complex on the membrane and in the cytosol (Doughman *et al*., 2003; Tolias *et al*., 1998). In this complex, membrane-associated Rac activates PIPKIα to produce a localized PtdIns(4,5)$P_2$. Platelet-derived growth factor (PDGF)-induced membrane ruffle formation is augmented dramatically by increasing ratios of active Rac to PIPKIα in MG63 cells, indicating that the localized association of PIPKIα with Rac in membrane and the signaling events of activated Rac are crucial in localized actin remodeling to form cell membrane ruffles (Doughman *et al*., 2003).

As an extension of these observations, the role of specific PIP kinases in the regulation of cell migration is now partly being clarified. There are several alternatively spliced variants of PIPKIγ in cells. Among them, two major variants, PIPKIγ635 and PIPKIγ661, which differ by a 26-amino acid C-terminal extension (Ishihara *et al*., 1998). The 26-amino acid C-terminal extension binds to talin and targets PIPKIγ661, but not PIPKIγ635, to focal adhesions (Di Paolo *et al*., 2002; Ling *et al*., 2002). These specific translocation of PIPKIγ661 allows for the generation of PtdIns(4,5)$P_2$ at focal adhesion in a spatial and temporal manner, which is identified to enhance the local association between integrin and talin (Martel *et al*., 2001). Talin is one of cytoskeletal proteins involved in connecting the integrin family of cell adhesion molecule with the actin cytoskeleton in focal adhesion. The interaction of talin to β-integrin enhances the affinity of integrin for its ligands and activates the integrin heterodimers (Tadokoro *et al*., 2003). PIPKIγ661 targeted to adhesions may modulate the integrin signaling and the adhesion formation through modulation by focal adhesion kinase, Src, and talin (Ling *et al*., 2002, 2003). These data suggest that PIPKIγ661 plays critical roles in modulating focal adhesion dynamics for cell migration.

Contrary to a robust accumulation of PtdIns(3,4,5)$P_3$ at the leading edge of migrating cells in response to a shallow gradient of external chemoattractant (Charest and Firtel, 2006; Janetopoulos *et al*., 2004; Wang *et al*., 2002), overall levels of cellular PtdIns(4,5)$P_2$ are relatively high and undergo only modest changes upon cell migration (Ling *et al.*, 2006). Nevertheless, local PtdIns(4,5)$P_2$ production is tightly regulated, both spatially and temporally, to execute the rapid adhesion turnover and cytoskeletal rearrangement in the leading edge of migrating cell. For example, local EGF stimulation induces a rapid co-translocation of PIPKIγ661 and talin to the leading edge and assembly into nascent adhesion (Sun *et al.*, 2007).

Integral membrane proteins such as E-cadherin are transported to and from the plasma membrane in lipid vesicles (Brett *et al.*, 2002; Cremona and De Camilli, 2001; Kirchhausen, 1999; Martin, 2001; Takei and Haucke, 2001). Strict regulation of vesicular trafficking pathway controls the amount of E-cadherin residing on the plasma membrane and is essential for modulating E-cadherin functions and adherence junction assembly (Bryant and Stow, 2004). In mammalian cells, trafficking and assembly of E-cadherin is controlled by diverse signaling events which include small G-proteins Rab and Arf6, p120-catenin, tyrosine phosphorylation, and ubiquitination (D'Souza-Schorey, 2005). Transportation of E-cadherin is controlled by the composition of cadherin complex as well as the vesicular trafficking machinery composed of multiple adaptor and signaling proteins (D'Souza-Schorey, 2005). Several lines of evidence suggest that endocytic E-cadherin trafficking is mediated by both clathrin dependent- and independent pathways (Le *et al.*, 1999). Both of these pathways depend on phosphoinositide signaling molecule, particularly, PtdIns(4,5)$P_2$. Endocytosis of E-cadherin requires clathrin lattice assembly and is controlled by a family of adaptors known as the clathrin adaptor-protein (AP) complex (Kirchhausen, 1999). AP complexes, such as AP-1, AP-2, AP-3, and AP-4 are important for recognizing signals for sorting of transmembrane proteins to endosomes and lysosomes. These AP complexes consist of several distinct proteins subunits which mediate endocytosis and sorting in epithelial cells (Gan *et al.*, 2002). Of these subunits, the $\mu$ subunit of AP complex contains the binding site for tyrosine- or dileucine-based cargo sorting motif (Bonifacino and Traub, 2003). Interaction of the $\mu$ subunit of AP-1B/AP-2 complexes with tyrosine sorting motif of cargo proteins is PtdIns(4,5)$P_2$-dependent process, which is crucial for membrane trafficking and endocytosis (Cremona and De Camilli, 2001; Honing *et al.*, 2005; Martin, 2001).

Local level of phosphoinositides is controlled by specific lipid kinases and phosphatases. However, the precise mechanism by which PtdIns(4,5)$P_2$ production is regulated to control these trafficking events has not been elucidated. Recent data demonstrate that spatial and temporal targeting of type I PIP kinases (PIPKIs) is a crucial mechanism for PtdIns(4,5)$P_2$ generation in controlling membrane trafficking and formation of adherence junction (Ling *et al.*, 2007). In MDCK canine epithelial cells, PIPKIγ661 co-localizes with the E-cadherin dimers through spatial and temporal targeting to cell adherence junctions. The translocated PIPKIγ661 modulates E-cadherin trafficking and adherence junction formation by localized production of PtdIns(4,5)$P_2$. PIPKIγ661 is now postulated to act as a scaffold molecule which combines E-cadherin and AP complexes together (Ling *et al.*, 2007).

***Conclusion and perspective.*** The current concept suggests that PIP kinases are translocated to specific subcellular compartments in spatial and temporal manner by interaction with specific targeting module, resulting in targeted production of PtdIns(4,5)$P_2$ and modulation of site-specific function (Santarius *et al.*, 2006). Thus, PIPKIγ661 targeting to focal adhesions by an association with talin results in spatially generated PtdIns(4,5)$P_2$, which regulates EGF-stimulated directional cell migration. In addition, a spatial targeting of PIPKIγ661 to E-cadherin dimers in cell adherence junction suggests a significant role of PIPKIγ661 in controlling dynamics of cell adherence junction and E-cadherin endocytosis, which is necessary and crucial in modulation of EMT process (Fig. 1). Cell stress caused by diverse toxicants can induce alterations in well established EMT-related cell signaling pathways such as changes in the expression of transcription factor Snail and related target genes. Moreover, it is conceivable that toxicants may influence on phosphoinositide signaling pathways or the targeting factors for PIPKIγ661 translocation to focal adhesion or cell adherence junctions (Fig. 1). Col-

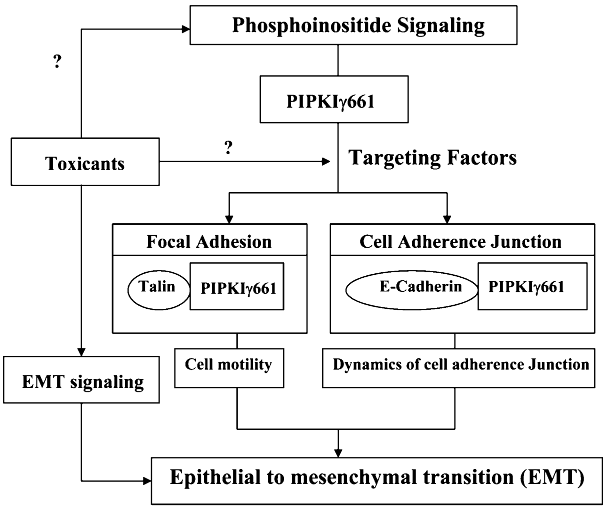

**Fig. 1.** Phosphoinositide signaling and EMT.

lectively, current review will provide us putative topics for investigating novel modes of action of EMT-inducing toxicants in terms of phosphoinositide signaling pathways.

## ACKNOWLEDGEMENT

This work was supported by Korea Science and Engineering Foundation grant RO1-2005-000-10596-0 funded by the Korean government
